# Going or Returning to Nature? Wild Vegetable Uses in the Foraging-Centered Restaurants of Lombardy, Northern Italy

**DOI:** 10.3390/plants13152151

**Published:** 2024-08-03

**Authors:** Naji Sulaiman, Dauro M. Zocchi, Sara Bonafede, Chiara Nanni, Renata Sõukand, Andrea Pieroni

**Affiliations:** 1 University of Gastronomic Sciences, Piazza Vittorio Emanuele II 9, 12042 Pollenzo, Italy; d.zocchi@unisg.it (D.M.Z.);; 2 Department of Environmental Sciences, Informatics and Statistics, Ca’ Foscari University of Venice, Via Torino 155, Mestre, 30170 Venezia, Italy; 3 Department of Medical Analysis, Tishk International University, Erbil 44001, Iraq

**Keywords:** ethnobotany, wild food plants, Lombardy, Italy, chefs, gastronomy

## Abstract

Wild vegetables (WVs) have been an essential source of human nutrition since ancient times. Foraging is a millennia-old practice that has gained more attention recently and is becoming fashionable, especially in restaurants in urban areas, as they attract customers who see WVs as an innovative sensory element and specialty food. Some cooks have used very few WVs for decades, but most chefs have only recently introduced them in their modern restaurants. Our study aims to have a deeper understanding of the diversity of WVs used by restaurants in the Lombardy region in Northern Italy and to know how they are introduced onto different menus, as well as the source of knowledge and the innovation paths related to the use/introduction of WVs in the selected sample of restaurants. Semi-structured interviews were conducted with 15 restaurant managers, chefs, and their professional foragers in the Lombardy region in Northern Italy in 2022; fifty-four wild plant taxa were recorded to be used in the considered restaurants. The collected data were analyzed to understand the current situation and the potential developments of this practice by exploring the reasons/motivations that underpin the inclusion of WVs in restaurants. A broad spectrum of restaurants was considered to evaluate the potential differences in handling and sourcing these ingredients. The results demonstrated that this trend has mainly been driven by attempts to revitalize traditional cuisines and to generate a positive impact on health, but the actual culinary preparations based upon WVs are often original and remarkably diverge from the Italian food ethnobotanical heritage. Moreover, concerns related to the environmental sustainability of these practices have been addressed.

## 1. Introduction

Wild vegetables (WVs) are those plant species gathered in the wild to be eaten as food or consumed as drinks [1,2]. WVs have been an important source of nutrition since ancient times, and they have played a crucial role in human survival for a long period of history [3,4,5]. Archaeological studies demonstrate that foraging wild plants occurred as early as 10,000 B.C. during the Early Pre-Pottery Neolithic age [6].

Due to several social and cultural factors, foraging has changed over time and space [7,8,9]. Wild foods still play a key role in some societies’ diets, while others have abandoned using these resources [7]. The use of WVs has been linked in several studies to famine and war-time food [4,5,10], and often perceived from the 1970s onward, at least in the Western European realm and the Levant, especially by younger generations, as a non-prestigious food [3,11]. The decrease in the consumption of WVs, which is linked to an increase in the availability of industrial food production and a detachment of people from nature, has led to the erosion of their associated traditional food and ecological knowledge [1].

Despite these negative trends, from the second half of the 20th century, renewed interest in WVs arose in several European countries [7,12,13], on a large scale, primarily in Northern and Central Europe and later also in the South of the continent. In the 1970s, the first interest in WVs by mass media rose with the publication of books such as “The Handbook of Edible Wild Plants” by Euell Gibson [14] and “Food For Free” by Richard Mabey [15].

In the past two decades, the practice of “foraging” WVs has gained immense popularity in the restaurant sector, especially among fine-dining restaurants, due to the remarkable role played by the Nordic Food Movement and, most notably, René Redzepi, one of the key ambassadors of this trend [16,17,18].

Traditional and non-traditional wild plants are increasingly used in the West’s restaurant sector, combining ancient practices with innovative cooking techniques. However, the first prodromic interest in wild food plants was anticipated in Europe in France: in 1963, Jean and Pierre Troisgros created a dish that symbolized the nouvelle cuisine made of salmon escalope with *Rumex acetosa*. This example shows how high-end restaurants changed the perception of this product from being a famine food to a delicacy and an element to innovate their culinary offering [19].

At the end of the nineties of the past century, WVs emerged as the prominent pillar of cuisine by a few French chefs, such as Michel Bras, Jean Marie Dumaine, and Marc Veyrat, active in France, Switzerland, and Germany [20,21,22].

The link between food and the local landscape is through the inclusion in restaurants’ menus of ingredients and dishes that represent the terroir and the taste of a place [23,24,25,26].

Most people perceive foraging, i.e., collecting food plants from the wild, as a sustainable practice that celebrates local produce, promotes well-being, and creates custodians of natural habitats [27]. Huang and Hall [28] claimed that food foraging can benefit the locality in terms of local seasonality, culinary culture, and biodiversity.

However, a careful literature study reveals essential concerns, especially regarding gathering specific species, which have gained the attention of the markets and, therefore, become often overexploited [29]. In a recent study among Nordic foragers, the awareness about bio-conservation and sustainability issues seems to be robust [30].

WVs have been studied in the past decade, especially in developing countries, as they are an essential source of nutrition for communities’ diets [31,32]. Meanwhile, Western societies focus more on the role of wild food plants in the rural and sustainable development of peripheral areas [33,34].

The restaurant sector in the Global North is now using WVs often as they are promoted as a sustainable ingredient, usually locally sourced, widely available, and fresh [18]. Some WVs have been used by renowned international chefs in signature dishes. In the restaurant sector, the use of WVs (and more generally of local ingredients) has been pointed out as a strategy to strengthen the embeddedness of the restaurant in the local foodscape, to provide an ethical and environmental footprint to the restaurant, and to innovate the culinary offering of the restaurant [35]. As highlighted in academic studies and the media, the role of chefs as explorers and sustainability educators has emerged, as the examples in the robust corpus of literature show.

An increasing number of studies have delved into issues associated with the evolving patterns of contemporary foraging, such as those related to recreational activities [12,36,37,38] and the factors pushing this practice, as well as within the context of food-related activities such as tourism [39,40] and, to a lesser extent, the restaurant sector [41,42]. However, limited attention has been directed toward examining the dynamics that drive the incorporation of WVs in restaurants. Furthermore, there is a dearth of exploration into the primary implications of this phenomenon in terms of innovation trajectories and sustainability issues related to this emerging trend.

To partially fill this gap, we focused on the trend of foraging in restaurants in the Lombardy region (Northern Italy), one of Southern Europe’s most dynamic and wealthiest areas.

We specifically aimed to:

- Record the diversity of utilized WVs and their dishes in selected restaurants in Lombardy and their culinary uses;
- Assess the main differences in the use of WVs according to the type of restaurants and their geographical location.

## 2. Results and Discussion

### 2.1. Wild Vegetables Used in Restaurants of Lombardy

Table 1 shows wild vegetables reported by our interviewees. In total, 54 taxa were documented with different frequencies, belonging to 31 plant families. Asteraceae, Apiaceae, Aspargaceae, Boraginaceae, and Caryophyllaceae were the most represented plant families. Among the recorded species, *Allium ursinum*, *Borago officinalis*, *Humulus lupulus*, *Taraxacum officinale*, *Oxalis acetosella*, and *Urtica dioica* were our informants’ most frequently mentioned and used WVs.

Our results demonstrate a relatively high diversity of WVs compared to some previous ethnobotanical studies on WVs in Italy [43]; this could be attributed to the different origins of our study participants, which resulted in more diverse traditions and knowledge related to WVs. Previous studies from Italy have also shown that some of the highly reported species in our study (VC) are important species in many areas all over the country (e.g., *Borago officinalis*). A comparative study on wild food plant consumption in 21 local communities in Italy demonstrates that there are plants species that are common in various Italian regions (such as *Asparagus acutifolius*, *Reichardia picroides*, *Cichorium intybus*, *Foeniculum vulgare*, *Sambucus nigra*, *Silene vulgaris*, *Taraxacum officinale*, and *Urtica dioica*) [44,45]. All of these species (except *Reichardia picroides*) were reported in our study.

Ghirardini et al. [45] found the dominant botanical family for the top 15 quoted wild food plants in Northern Italy to be Rosaceae, while it is Asteraceae in Southern Italy. Our study highlights Asteraceae as the dominant family, which may be interpreted by our focus solely on WVs.

Most reported WVs were harvested for their leaves, shoots, and flowers but rarely for their roots. Some chefs are also using new parts of WVs and creating innovative recipes.

Some of our reported species can also be found cultivated in home gardens (e.g., *Tropaeolum majus* and *Foeniculum vulgare*); however, our study participants reported that they collect all of these species from the wild (unless mentioned otherwise).

### 2.2. Recipes with WVs

Traditionally, wild plants have been used for various preparations, both cooked and raw, depending on the season and the time they are gathered. However, they often must be blanched for a few minutes to be more digestible and palatable. Namely, they are used to prepare omelets, soups, pasta fillings, and quiche-like savory pies.

**Figure 1:**
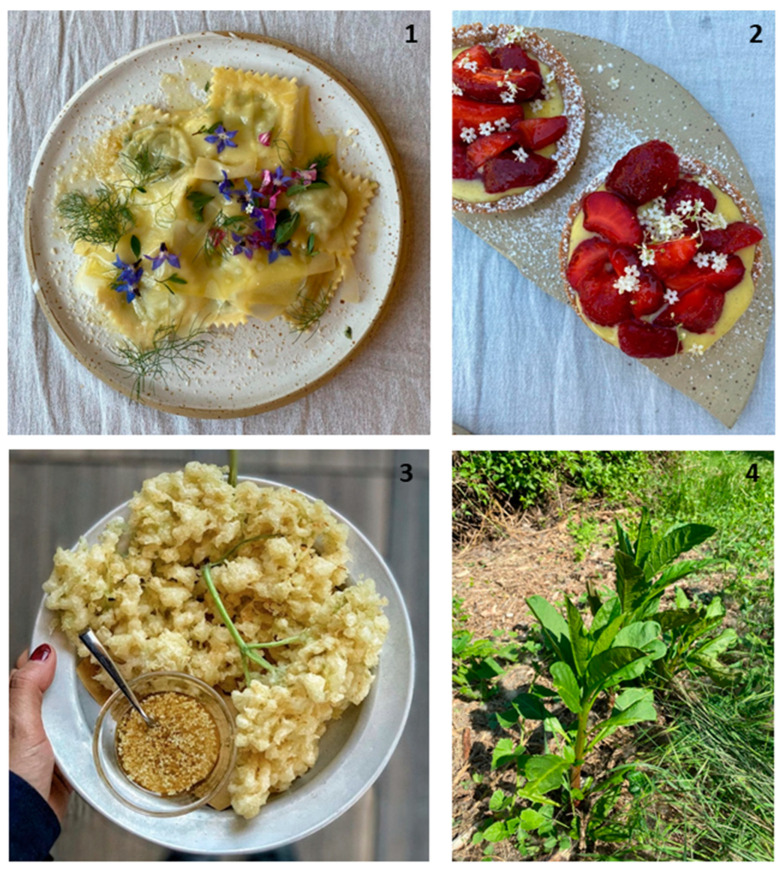
Wild vegetable-based dishes. **1**: Tortelli filled with *seirass (ricotta cheese)* and *Taraxacum officinale,* garnished with *Borago officinalis* and *Sambucus nigra* flowers and leaves of (cultivated) fennel. **2**: Pastry prepared with strawberries and *Sambucus nigra* flowers. **3**: Deep-fried flowers of *Sambucus nigra.* **4**: *Phytolacca americana* where young shoots are used for dessert. (Photo credits: S.B. and C.N.).

One of the most used plants in Lombardian tradition is *Taraxacum officinale*, which was used by our respondents both to recreate traditional recipes (using the leaves), such as risotto with *T. officinale* (*risotto al tarassaco*), filled pasta with ricotta and *T. officinale*, and salad with *T. officinale* and boiled eggs (Figure 1). However, to avoid food waste, the roots can also be used as a substitute for coffee by toasting them or as a starter by serving them fried. More innovative uses of this plant can be seen in high-end restaurants, where flowers of *T. officinale* are preserved in syrup with bittersweet spruce shoots syrup with chocolate and myositis leaves. In other cases, it is fermented with legumes and cereals to create crumbs added on some first courses.

*Urtica dioica* is mainly used in traditional first-course dishes, such as fresh pasta, soups, or gnocchi, which are often prepared using *T. officinale, Allium ursinum,* mint, *Silene vulgaris*, *U. dioica*, and sage as well. According to some study participants, it tastes similar to arugula and can be consumed fresh when collected at the beginning of spring.

*Rise Vartis* (rice with young shoots of *Humulus lupulus*) is another traditional recipe. Many interviewees mentioned that such a dish would be eaten at their grandparents’ house; according to them, the wild hop shoots resemble tiny asparagus shoots and have a slightly aromatic but delicate taste.

Some of our reported species have different other uses across Italy and neighboring countries. For example, in addition to the frying method of preparation, *Muscari comosum* is cooked and pickled to reduce its bitterness, it is also preserved in vinegar and eaten as an appetizer with several kinds of food [44,46].

“Frittelle di sambuco” (fritters made with the inflorescences of *Sambucus nigra*) was another typical sweet of childhood time for most of the study participants. *S. nigra* was also one of the most mentioned species, mainly used to produce syrups. However, in high-end restaurants, elderflowers are lacto-fermented and used as pastry ingredients. On the other hand, *A. ursinum*, the leaves are widely used to prepare a pesto sauce or to garnish first courses such as pasta. *Oxalis acetosella* is commonly used in salads and in the creation of innovative recipes.

### 2.3. Relationship between the Study Participants’ Background and the Use of WVs

Out of the fifteen interviewees, only three were not originally from Lombardy, with one from Sicily in the south of Italy, another from Veneto in the northeast of Italy, and the third from the United States of America. The chefs’ recipes reflected their place of origin and the area in which the restaurant is located as they use WVs that are locally foraged.

Our results show that some chefs used plants typical of their traditional area of origin, which does not necessarily match Lombardy’s traditional domestic cuisine. For instance, the majority of the wild vegetables used by a Sicilian chef, the owner of a restaurant in Milan, are typical for the South of Italy (e.g., *Silybum marianum, Foeniculum vulgare,* and *Cichorium intybus*), who prepared some dishes such as fava beans puree and *Taraxacum officinale* and pasta with mussels and *F. vulgare*. However, the participant stated that when he moved to Milan, he began to integrate new species into his cuisine that he had never used before, such as *Borago officinalis*, despite the use of this species being common in both Northern and Southern Italy [45]. Meanwhile, another study participant (a chef), originally from Veneto, also reported that he often offers traditional recipes from his region (Veneto), such as wild hop (*Humulus lupulus*) risotto, which is also typical in Lombardy due to the geographical closeness.

It is interesting to notice how more traditional Lombardian restaurants tend to offer more first courses, such as pasta with wild vegetables; for example, one of the interviewed chefs offers gnocchi (*Malfatti*, a traditional Lombardian dish) made with *B. officinalis*, *U. dioica*, Grana cheese, and *Portulaca* and egg pasta with *H. lupulus*. One of the sampled restaurants, located in the province of Varese, also has on its menu *T. officinale* and *U. dioica*, which can be found in the following dishes: “Pasta filled with *B. officinalis*, perch fish, saffron (*Crocus sativus*) and edible flowers” and spelt gnocchi with *Urtica dioica* and hazelnuts (*Corylus avellana*).

Meanwhile, high-end restaurants tend to be more innovative in using WVs. One such restaurant is located in a small village in the Como province; it offers, for example, cow meat with sorrell and raspberries, risotto with watercress and fermented trout, and snails with *Allium ursinum* and the mushroom *Auricularia heimuer*.

### 2.4. Foraging and Study Participants’ Knowledge of WVs

Restaurant chefs and staff managed to forage on their own if situated close to nature, and 70% of chefs and restaurateurs reported that they forage wild vegetables on their own. On the other hand, restaurants located more in the city center obtained wild plants gathered by professionals or retired women who had the knowledge to engage in this activity. However, even when chefs were buying wild plants from an external party, they showed deep understanding and interest, explaining that, when possible, they would forage in areas closer to the mountains.

Regarding the harvesting practices, the chef and owner of a fine-dining restaurant in the Como province explained that he maintains a constant presence, with the entire kitchen staff joining the foraging activities. He also imparts knowledge to newcomers on what and how to collect, eventually entrusting them with solo collection tasks for more straightforward items like *U. dioica* and *Robinia pseudoacacia*. While time-intensive, the team’s self-sourcing of wild plants allows for selection of more sophisticated specimens and fosters a cohesive experiential bond among team members. However, as chefs are usually busy with other tasks in the kitchen, they often ask the staff to gather the plants needed for the menu.

The restaurants in Milan stated that they rarely have time to forage and, therefore, ask independent foragers to provide them with the wild species they need. Most WVs are collected in the spring, between April and May, when the temperatures are not too elevated. However, due to climate change, the interviewees stated that the season of each plant is slowly shifting. Some species, such as *U. dioica*, *T. officinale*, and *H. lupulus*, grow both on plains and at higher altitudes. Therefore, they can be collected at different times and have a longer mountain harvest season.

A thorough examination of interviews with chefs and foragers unveiled a compelling interconnection among professional training, avenues of knowledge acquisition, and individual motivations that influence the incorporation of wild plants in restaurant practices.

Participants’ statements consistently reference family traditions as the primary source of knowledge acquisition regarding the recognition and use of wild herbs. Quotes from participants underscore the generational transmission of such knowledge, emphasizing the pivotal role played by parents and grandparents in this process.

Most restaurants outside of urban areas inherited the business from their parents or grandparents and all the knowledge related to this practice due to their close contact with nature. While interviewing some chefs who owned or worked in restaurants in rural areas, it emerged that it was an integral part of people’s lives to gather WVs. Many recipes are still the same tradition that represents the territory and gastronomy of Lombardy, being handed down from generation to generation. Still, nowadays, they are appreciated by customers, and this makes these restaurants unique. “*I inherited a cookbook from my mother, made of recipes that she gathered from friends, relatives and public libraries*”, one participant reported. However, some participants reported that they have observed a change in traditional knowledge of WVs over time and space. In this context, Ghirardini et al. [45] proposed a hypothesis that the erosion of traditional knowledge in Southern Italy on wild vegetables is slower than in Northern Italy and also the likelihood that Southern Italians’ have a higher appreciation of wild vegetables that have a strong and bitter taste.

One of the interviewees specified that she often uses alternative tools to recognize some plants she is not sure about by using software applications like “Herbarium”, which is an application that provides a detailed description of a specific species just by taking a picture of it. Moreover, it allows you to save the previous plants you have researched. Only two interviewees, both chefs in experimental haute cuisine restaurants, reported acquiring their knowledge primarily within a professional setting.

### 2.5. Motivations for and Perspectives of WV Use

The interviews shed light on compelling motivations driving chefs to incorporate wild plants into their culinary offerings. Three primary themes emerged: the expression of local territory, the desire to uphold tradition, and the inclination to experiment and innovate from both a sensory and raw materials perspective. A notable aspect was the age-dependent prevalence of the desire to experiment and innovate, with exclusively younger interviewees, those under 45, expressing this motivation.

Most of the study participants emphasized the significance of their geographical location, considering it a responsibility and duty to reflect the endemic WVs found in the surrounding environment in their cuisine. The usage of wild plants is inherently tied to the natural setting, expressing a commitment to showcasing the region’s diversity beyond conventional culinary pairings. One of our participants reported: “*The first reason is absolutely the place in which we find ourselves; here in nature, there are many endemic herbs, and it seems only right to use them*”.

Even in urban settings, as in the case of a restaurant in Milan, participants acknowledged the significance of their location in influencing their choice to incorporate spontaneous plants into their cuisine. For some chefs, especially those who inherited the business from their parents, using WVs has been a common practice connected to the culinary tradition of their families and communities. However, this activity changed from being related to survival to a recreational one as a way to reconnect to nature.

“*We started gathering wild plants 25 years ago. Our purpose was to add value to our land by using local products that are part of our gastronomic tradition*”, one of the participants reported.

All restaurants indicated that communication on using WVs occurs through service staff who are knowledgeable about the ingredients and their origin. In some cases, pictures of the plant are shown to customers to give a better overview of the species used in a particular dish. However, only one restaurant stated that it uses the local and scientific names of WVs on the menu. Meanwhile, another chef reported that they do not like to stress the fact that they use uncultivated plants but prefer the dish to “talk for itself”, as food needs to be enjoyed first.

Even though such communication is not accessible, one chef and owner of a restaurant in the Como province explained his perspective on this issue:

“*On social media, we communicate the use of wild plants simply. Our goal is to explain our philosophy, emphasising that we use ingredients that we gather in the surrounding area. Meanwhile, I try to go more in-depth when I talk to a client interested in this topic*”.

A few restaurants reported that they use social media to communicate the use of WVs and their story, which can be very powerful in educating customers. “*We tell the story of each plant and the origin of their local names both in the presence and through social media. We also organise experiences in the wild to show the plant to our customers. However, we believe that telling a story in person is more effective*”, a chef and owner of a traditional restaurant in Pavia province reported.

Communities’ perception of wild vegetables has changed with time, and until the 1960s, it was associated with a subsistence lifestyle. Moreover, the standardization and modernization of food production has accelerated the loss of this practice and knowledge [47]. Thanks to movements like the New Nordic Cuisine and Slow Food, traditional practices are being valued again, while chefs are determining new “transnational food identities” [48]. In general, chefs noticed that their customers are curious and knowledgeable about the wild foods they offer. In the case of long-standing customers, they are familiar with the ingredients used and sometimes ask the restaurant for some wild plants to take home. More often, customers become informed beforehand or ask several questions to gain knowledge about the story related to the specific variety. “*A while ago, people would criticise us, saying that we only served grass at the restaurant; meanwhile, nowadays, people enjoy and appreciate them*,” one chef reported. As WVs were usually related to periods of hunger until 15 years ago, their use in restaurants was not seen in a good light. However, it is now perceived as an element of innovation.

Respondents agree that sustainability in foraging is a significant issue. If some plants become too prevalent in gastronomy, they might be overharvested. These concerns especially arise when roots are harvested, preventing the plants from growing again, particularly with perennial species. “*Having become fashionable, foraging faces a risk of no longer being sustainable. As long as I am in my small town where few people collect herbs, it is fine; however, when it becomes a trend, there is a serious concern*”, one participant reported. Considering these sustainability concerns, we checked our reported species with the IUCN Red List and found no critically endangered species [49].

Consuming wild vegetables has many benefits, as they contribute to a healthy diet, promote local economies, and improve landscape multifunctionality [50]. Some of our documented species have been previously reported for consumption as “food-medicine”, such as *A. ursinum*, *F. vulgare*, and *C. intybus* [8]. Overall, they have high economic potential for local people and health benefits, as they contain antioxidants, vitamins, and minerals, which are important, especially in the diets of communities from developing countries. They can also be used as a medicine, preventing chronic diseases such as obesity and type II diabetes [51]. Moreover, as they are uncultivated species, they have no cost and preserve tradition.

Our participants’ perspectives on WV use mainly focused on environmental impact, health considerations, and the tension between fashion and tradition. While most respondents touched upon all three subjects, they presented varied arguments. From an ecological standpoint, foraging was viewed as having positive and negative aspects based on its practice. Harvesting was often deemed sustainable as it contributes to biodiversity preservation, aids pollination, and avoids using irrigation, pesticides, and fertilizers. However, concerns were raised about negative impacts when harvesting is carried out improperly or disrespectfully. Respondents emphasized the importance of responsible foraging to prevent environmental overexploitation. The health benefits of wild plants were highlighted by many, with ten out of fifteen respondents expressing satisfaction that foraging has become popular due to its potential health advantages. This is reflected in one of the participants’ statements: “*There is a reason why our grandparents lived to be 90 y.o.*”. Some mentioned that certain herbs can be more nutritious than fruits and vegetables when harvested correctly. Despite the positive outlook, there was a shared concern among some respondents about inexperienced individuals mistakenly consuming toxic or contaminated plants. The theme of the contrast between fashion and tradition was prevalent in eleven interviews. While some celebrated the continuation of a tradition dating back to “the dawn of time,” others expressed discontent with foraging becoming a trendy activity. Critics argued that some people engage in foraging solely for fashion, misrepresenting cultivated plants as wild and diluting the practice’s authenticity:

*“In Italy, in difficult times, foraging was done out of necessity. Now, it has become a luxury. Many say they forage, but in reality, they buy. Just because it’s edible, an ingredient is not necessarily good, like fermentation”*

one of our study participants reported.

However, comparing the quoted culinary preparation with the *Italian Compendium of Gastronomy* by the Italian Touring Club, published in 1931 [52], and with the survey conducted in Lombardy half a century ago by Nino Arietti [53], a crucial finding emerges: more than half of the contemporary restaurants’ culinary preparations are not part of the traditional heritage of Italian cuisine (see uses reported in italics in Table 1). This is an essential outcome since the Traditional Knowledge (TK) regarding WVs that the study participants seemed to indicate as one of the drivers in using these wild ingredients has possibly been acting as a nostalgic trigger for the practice of foraging but less for the acting of processing/cooking WVs, as a lot of innovation seems to be currently embedded into the dishes that the considered restaurants offer.

This nostalgia was represented by a few participants’ statements, such as:

“*I started foraging with my grandfather as a kid. I remember going for walks in nature and collecting wild hops*”

(conveyed by a forager born in northern Italy).

As for future developments, those who expressed opinions unanimously predicted a decline in the foraging trend. However, this decline was seen positively, with the belief that it would filter out those who engage in foraging solely for trend-following purposes, leaving committed and conscientious practitioners.

Two interviewees anticipated that foraging would persist mainly in regions with historical roots, emphasizing its connection to local traditions. Respondents hoped for increased awareness and responsible practices, anticipating a shift from trend-driven foraging toward a more thoughtful and professional approach.

Some of our study participants highlighted an increase in the time required for WV preparation due to inconsistencies in the availability of WVs, which consequently necessitated designing more flexible dishes and menus. *C. intybus* was mentioned as one of the most complex plants to clean and work with, as it always comes in different shapes and requires a lot of preparation. Some of our study participants reported that using WVs does not mean dominating nature but being wholly dependent on it. “*You cannot predict the availability of a certain product, it depends on the weather conditions and if it was a good year*”, one chef stated. There is a paradigm shift, as chefs can no longer decide exactly which ingredients to use on their menus, but have to wait until they receive the ingredients, and then they create the menu according to what they have.

## 3. Materials and Methods

### 3.1. Study Area

This study was conducted in the Lombardy region, situated in the Northern part of Italy. Moreover, in Lombardy, 25% of the land is characterized by protected areas, 24 regional parks, 3 natural reserves protected by the state, 67 protected by the region, and National Stelvio Park (the biggest national park in Europe).

Lombardy is located between the mountain Alpine range and the Po River, which creates the perfect conditions for rich flora and fauna. It is characterized by an extended plain (Pianura Padana), which covers 47% of the land; 41% of the land is occupied by mountains (Prealpi Lombarde, Alpi Orobie, Alpi Lepontine, and Alpi Retiche); and 12% by hills [54]. Lombardy’s extensive territorial expanse, diverse morphological configurations, and the presence of lakes and rivers correspond to a remarkable climatic variety and, consequently, plant species diversity. The region boasts a total of 3220 floristic species, accounting for just under 50% of the national floristic diversity [55], including 61 endemic species, with 48 exclusive to Lombardy [56].

### 3.2. Field Study

**Figure 2:**
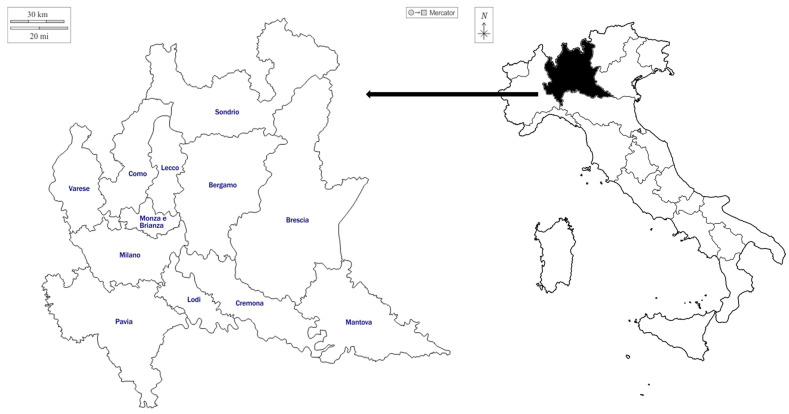
Study area map. Key locations: Bergamo (Foppolo), Como (San Fermo della Battaglia), Cremona (Stagno Lombardo, Lodi, Milano (Milano, San Bovio, San Felice), Sondrio (Madesimo), and Varese (Cuasso al Piano).

Fieldwork research was conducted in Lombardy during spring 2022 (Figure 2). General scouting of restaurants in the Lombardy region was performed using web platforms and restaurant guides, including the Italian Michelin Guide and the Slow Food Osterie d‘Italia Guide. In this context, the descriptions of the restaurants in the sources, as mentioned earlier, were reviewed to identify any specific mentions of WVs. Furthermore, by analyzing the websites and social media pages of these restaurants, we were able to outline the culinary offerings in general terms and gain a better understanding of the role and relevance of WVs on their menus.

Based on the described criteria, we prepared a list of 25 restaurants in seven Lombardy provinces. Those restaurants were using WVs substantially and consistently rather than occasionally. Subsequently, we contacted the restaurants to explain the research aims and to arrange interviews. Of the 25 restaurants, 11 (managers or chefs) agreed to participate in the study. Additionally, to gain a broader perspective of WV use in restaurants, we interviewed four professional foragers who gather WVs for a few of these restaurants (Table 2).

Both high-end/modern and traditional/popular restaurants were considered to ultimately have a better overview of the use of WVs in different restaurant settings and different territorial contexts, i.e., rural and urban areas.

The interviews took place between April and May 2022. Fifteen in-depth interviews were conducted with restaurant managers, chefs, and a few of their foragers. Half of the interviews took place in the urban area of Milan, as the restaurant density is higher and many facilities have implemented the use of WVs in their cuisine. In contrast, the rest of the restaurants were located in the most remote areas, in the provinces of Milan, Sondrio, Bergamo, Pavia, Varese, and Cremona, as more representative of the Lombardy cuisine related to the use of WVs.

The questionnaire was designed to gain a precise understanding of WV use in the restaurant sector. We applied the same questionnaire to the 15 study participants. However, specific parts of the questionnaire were discussed more in-depth based on the interviewee’s profession; for instance, questions to the foragers were mainly focused on the foraging activity rather than the culinary ones and vice versa with the chefs.

Participants were firstly asked to list all of the WVs they forage and use in the restaurants. They were requested to report the plant’s local names, parts used, seasonality, and culinary preparations. Additional inquiries were directed toward exploring the ethnobotanical knowledge sources of our study participants and the motivations behind incorporating wild plants into their cuisine. Informants were also asked to report the advantages and disadvantages associated with WV use in cooking, and how they market their WV-based dishes to customers. Discussions with our study participants delved into the current trend of foraging and its evolving role in the hospitality industry.

The Code of Ethics of the International Society of Ethnobiology was followed during this research, as every interviewee was previously notified of the aims and goals of this project [57]. Oral informed consent was required in order to be recorded during the interview and to publish the results of the research. Following the ethical obligations in ethnographic research, the results will be made accessible, maintaining respectful and ethical behavior [58]. Interviews were conducted in Italian; most quoted wild plant specimens had been collected, identified, and deposited at the UNISG Herbarium in previous ethnobotanical fieldwork conducted by some of the authors in NW Italy [59]. The non-collected species were identified using local names and plant descriptions following a previous (food) economic–botanical survey in the Brescia area, Lombardy [53].

The study was largely based on a qualitative analysis of the collected data. The interviews and the field notes were transcribed and analyzed using NVivo qualitative data analysis version 12.5.0 [60]. Data were analyzed using quality content analysis [61] to explore the main motivations, perceptions, and issues behind the inclusion of WVs in the culinary offerings of the selected restaurants. We calculated the frequency of plant citations based on the number of mentions by the interviewees.

Data gathered in the survey were compared with those of the *Italian Compendium of Gastronomy* by the Italian Touring Club, published in 1931 [52], and with the economic–botanical field survey conducted in Lombardy half a century ago by Nino Arietti [53].

The limitations of this study were mainly represented by the relatively small number of restaurant chefs who agreed to be interviewed on the studied topic. However, we tried to mitigate this limitation by conducting in-depth interviews with all of the study participants. Another limitation was related to the very scarce academic literature on the past use of wild plants in lowland Lombardy’s cuisine since this practice, with the exception of the Alpine portion of the region, was possibly abandoned earlier than in other Italian regions or was not the object of ethnobotanical/traditional foraging studies during the past three–four decades, as has happened in other Northern Italian areas.

## 4. Conclusions

The results of this study show that foraging is a practice that is part of the tradition of every participant. Even if it is increasingly becoming a trend, the respondents showed real interest in the topic and detailed technical knowledge. It is a return to the roots, which can be positive by creating awareness about wild foods but could also lead to the overharvesting of specific species considered trendy in restaurants. Climate change also plays a vital role in the growth and use of these plants. Moreover, as more chefs move to cities such as Milan, combining traditional recipes with wild vegetables from different Italian regions creates new recipes that might become part of the Lombard tradition. Most restaurants in remote areas of Lombardy showed some relationship to tradition and old recipes, while more young and innovative urban chefs are developing new cooking techniques with WVs; however, more than half of the overall recorded culinary preparations are not part of the traditional heritage of Italian cuisine.

Overall, many have mentioned how foraging is an activity that helps them reconnect with nature and themselves. By cooking with wild foods, chefs cannot control directly which plants they will have on their menu, but they have to respect nature’s cycle and cook with what ingredients are available. The COVID-19 pandemic increased the interest of chefs and customers in wild foods. Many foragers also built a strong community on social media by sharing their knowledge and creating curiosity, even among people unfamiliar with WVs.

Future research trajectories could more closely examine how restaurants can impact customers’ and citizens’ relationships with nature and wild foods.

## Figures and Tables

**Table 1:** Table of recorded WVs used in the selected restaurants in Lombardy.

Scientific Name	Botanical Family	Common Name(s)	Part(s) Used	Culinary Preparations (Italics Refers to Traditional Lombardy Uses)	Frequency of Quotation
*Achillea millefolium* L.	Asteraceae	Achillea	Flowers and leaves	*Seasoning grappa*	C
*Aegopodium podagraria* L.	Apiaceae	Girardina	Flowers	Catfish with a mix of herbs	R
*Alliaria petiolata* (M.Bieb.) Cavara & Grande	Brassicaceae	Alliaria	Leaves and flowers	Decoration, dried and added on tomato sauce	C
*Allium ursinum* L.	Amaryllidaceae	Aglio orsino	Leaves and flowers	Pesto, *risotto*, stuffing for tortelli (stuffed pasta) with ricotta, *seasoning snails*	VC
*Arctium lappa* L.	Asteraceae	Bardana	Leaves and stems	Wrapped around a filling, blanched with garlic	R
*Asparagus tenuifolius* Lam.	Asparagaceae	Asparago selvatico	Shoots	Soups, *omelettes*, *and risotto*	C
*Bellis perennis* L.	Asteraceae	Pratoline	Flowers	Garnishing various preparations	R
*Betula pendula* Roth	Betulaceae	Betulla	Lymph	Syrup, wine, and desserts	R
*Borago officinalis* L.	Boraginaceae	Borraggine	Leaves and flowers	Salads and garnishing filled pasta	VC
*Calendula officinalis* L.	Asteraceae	Calendula	Flowers and leaves	*Salad* with miso and infused oil, infusions, and kombucha	C
*Cetraria islandica* (L.) Ach.	Parmeliaceae	Lichene	Thallus	Infusion	R
*Chenopodium album* L.	Chenopodiaceae	Farinello	The entire plant if young, otherwise the leaves	*Blanched*, *filling for pasta*, dried, *and gnocchi (malfatti)*	C
*Chenopodium bonus-henricus* L.	Chenopodiaceae	Paruch, Spinacio di montagna	Leaves	*Polpette* with cheese and *lasagne*	C
*Cichorium intybus* L.	Asteraceae	Cicoria selvatica	Leaves	*Blanched, as a side dish*	R
*Clinopodium nepeta* (L.) Kuntze	Lamiaceae	Nepitella	Leaves	*Infusions*	R
*Foeniculum vulgare* Mill.	Apiaceae	Finocchietto	Leaves and fruits	Pasta with mussels, *blanched*	C
*Equisetum arvense* L.	Equisetaceae	Coda cavallina, Equiseto	Fertile stems	Salads	R
*Gentiana lutea* L.	Gentianaceae	Genziana	Roots	*Seasoning grappa*	C
*Helichrysum italicum* (Roth) G.Don	Asteraceae	Liquirizia selvatica	Entire plant	Seasoning grappa	R
*Humulus lupulus* L.	Cannabaceae	Bruscandoli, Luppolo selvatico	Shoots	*Risotto, omelette, agnolotti* (stuffed pasta) with meat stew, soups, and *blanched and served with lemon*	VC
*Lathyrus oleraceus* Lam.	Papilionaceae	Pisello selvatico	Flowers	As a side dish of catfish	R
*Malva sylvestris* L.	Malvaceae	Malva	Leaves and flowers	Gnocchi (*malfatti*)	R
*Melilotus officinalis* (L.) Lam.	Fabaceae	Meliloto	Flowers	Infusions	R
*Melissa officinalis* L.	Lamiaceae	Melissa	Leaves and flowers	Pasta filling	R
*Muscari comosum* (L.) Mill.	Asparagaceae	Lampascione	Bulbs	*Fried*	R
*Myosotis arvensis* (L.) Hill	Boraginaceae	Non ti scordar di me	Aerial parts	Bittersweet syrup of spruce shoots with chocolate	R
*Nasturtium officinale* W.T.Aiton	Brassicaceae	Crescione	Aerial parts	Seasoning, esp. mussels and cabbage	R
*Oxalis acetosella* L.	Oxalidaceae	Acetosella	Leaves	Sorbet, infusions, and salad with almonds and watercress	VC
*Papaver rhoeas* L.	Papaveraceae	Rosole	Young aerial parts	Salads and *tortelli filling*	C
*Parietaria officinalis* L.	Urticaceae	Parietaria	Leaves	Soups and omelettes	R
*Phacelia tanacetifolia* Benth.	Boraginaceae	Facelia	Flowers	Decoration	R
*Phytolacca americana* L.	Phytolaccaceae	Fitolacca	Young shoots	Dessert	R
*Picea abies* (L.) H.Karst.	Pinaceae	Abete rosso	Shoots and pine cones	Pine cones in oil, bittersweet *syrup* with chocolate, and myosotis leaves	R
*Pinus mugo* Turra	Pinaceae	Pino mugo	Young pine cones	*Syrups*	C
*Plantago lanceolata* L.	Plantaginaceae	Piantaggine	Flowers and leaves	Salads, salt-cured, savoury pies, and pizza topping	C
*Portulaca oleracea* L.	Portulacaceae	Portulaca	Aerial parts	*Salads*	C
*Poterium sanguisorba* L.	Apiaceae	Pimpinella	Leaves	*Salads*, salads with bacon	C
*Primula vulgaris* Huds.	Primulaceae	Primula	Leaves and flowers	*Salads*, *blanched*, and garnish	R
*Robinia pseudoacacia* L.	Fabaceae	Acacia	Inflorescences	Garnish on pastries, *deep-fried*	C
*Rumex acetosa* L. and *R. acetosella* L.	Polygonaceae	Erba brusca, Acetosella	Leaves	Seasoning spaghetti with clams and black truffles	C
*Rumex obtusifolium* L.	Polygonaceae	Lapazio	Leaves	Rolls with trout filling	R
*Ruscus aculeatus* L.	Asparagaceae	Pungitopo	Shoots	*Fried with eggs* and mullet roe	R
*Salvia pratensis* L.	Lamiaceae	Salvia dei prati	Aerial parts and flowers	Syrups and infusions	R
*Sambucus nigra* L.	Caprifoliaceae	Sambuco	Flowers and fruits	Garnishing pastries; *infusions*, *syrups*, fried, tiramisù, lactofermented, jam, and panna cotta	VC
*Silene vulgaris* (Moench) Garcke	Caryophyllaceae	Silene	Leaves	*Salads, savoury pies*; with almonds, lemon, and black pepper; sesame *panelle* (chickpea flour fritters); with lettuce and sumac; accompanying grilled branches; as a side dish with black cherries and hazelnuts; crepes; and *risotto* with smoked butter	C
*Silybum marianum* (L.) Gaertn.	Asteraceae	Cardo mariano	Aerial parts and stems	In fava bean soups	R
*Stellaria media* (L.) Vill.	Caryophyllaceae	Pavarina	Leaves	Salads	R
*Symphytum officinale* L.	Boraginaceae	Consolida	Leaves	Salads, savoury pies, and pasta filling	R
*Taraxacum officinale* F.H.Wigg.	Asteraceae	Tarassaco	Leaves and roots	*Salad*, *filled pasta*, *coffee substitute (roots)*, risotto, grilled snails, fried roots, fermented roots with legumes and cereals, and *tortelli* with *seirass* cheese	VC
*Tilia* spp.	Malvaceae	Tiglio	Leaves	Salads and soups	R
*Tropaeolum majus* L.	Tropaeolaceae	Nasturzio	Leaves	Pesto with hazelnuts and almonds and risotto	R
*Urtica dioica* L.	Urticaceae	Ortica	Leaves	*Filled pasta*, *tagliatelle green coloring agent*, *risotto, soups, and gnocchi with herbs*	VC
*Veronica arvensis* L.	Plantaginaceae	Ederella	Leaves	Blanched, as a side dish	R

VC—very common: quoted by five or more study participants; C—common: quoted by 2–4 study participants; R—rare: quoted by one study participant only; *italics* writing in the “culinary preparations” column: traditional uses.

**Table 2:** Information on the study participants.

Participant Profession	Restaurant Name	Location
Cook/Chef	Radici Restaurant	San Fermo della Battaglia (Como)
Cook/Chef	Ristorante Cantinone	Madesimo (Sondrio)
Cook/Chef	Trippa	Milan
Cook/Chef	Ristorante Albergo Selvatico	Rivanazzano (Pavia)
Cook/Chef	K2 hotel e ristorante	Foppolo (Bergamo)
Cook/Chef	Cascina Lagoscuro	Stagno Lombardo (Cremona)
Cook/Chef	Pasta Madre	Milan
Cook/Chef	Erba Brusca	Milan
Cook/Chef	Lago Scuro	Stagno Lombardo (Cremona)
Manager	Molino del Torchio	Cuasso al Piano (Varese)
Manager	Erba Brusca	Milan
Forager	-	Milan
Forager	-	San Bovio (Milan)
Forager	-	San Felice (Milan)
Forager	-	Lodi—Parma

## Data Availability

The data supporting this study’s findings are presented within the article.
